# Book Review: The Psychology of COVID-19: Building Resilience for Future Pandemics

**DOI:** 10.3389/fpsyg.2021.744926

**Published:** 2021-09-13

**Authors:** Jing Zhang, Zhipeng Li

**Affiliations:** ^1^School of Marxism, Institute of Psychological Health, Hangzhou Dianzi University, Hangzhou, China; ^2^School of Medicine, Technische Universität Dresden, Dresden, Germany

**Keywords:** COVID-19, pandemic, uncertainty, risk, crisis, resilience

As the most prominent “black swan event” in 2020, COVID-19 discloses numerous social problems. Why is it so destructive? How to deal with such crises in the future? In *The Psychology of COVID-19: Building Resilience for Future Pandemics*, Dr. Joel Vos points out that the psychological factors behind the pandemic are vital. As a psychologist, philosopher, researcher, public speaker, and therapist, Vos considers what changes the lives of people dramatically is not COVID-19 itself but their perception and response. It seems that society cannot flexibly adapt to challenges and building a resilient society is the key to successfully dealing with the crisis.

This book includes eight chapters. Chapter1 (“The World Risk Society”) presents various uncertainties. Like what is the exact infection rate? What is the best way to deal with it? When will the global pandemic end? The influence of COVID-19 is undoubtedly extensive, but its indirect influence on the mental health of people may be more profound than its direct influence on physical health. If the biological impact is the first pandemic, then the psychological impact is the second one. Therefore, learning to live in harmony with uncertainties is necessary.

First of all, attitudes and expectations of people toward science should be adjusted (Chapter 2, “Natural Risks: Uncertain Science”). At the normal science stage, researchers share a particular paradigm. Whenever phenomena cannot match with predictions, regular scientists will try to eliminate puzzles while making as few changes as possible to the paradigm (Kvasz, 2014). However, science during the pandemic is abnormal, which cannot be reconciled with the theoretical assumptions of the current paradigm. It involves many ambiguities; it is irrational to expect that science can provide undisputed data and answers in this period. Still, a preference for certainty is instinctive, which is why people are so vulnerable to politics and the media during the pandemic.

Chapter 3 (“Social Risks: Uncertain Politicians”) starts with a statement: “Each pandemic is fundamentally political in nature.” Republican and Democratic supporters have different attitudes toward the social distance. Besides, when things go wrong, the government may shift responsibility for face and voters, such as blaming immigrants or other countries. Further, corruption, hyper-connection, and capitalization may aggravate the impact of the pandemic. Finally, biopolitics emphasizes humans as species, leading to monitoring, tracking, and isolation policies (Vali et al., 2020). Vos suggests that individuals need to develop critical thinking ability to reflect on the political system, while the collectives need to limit the imbalance of political power through the law to establish a more flexible political system.

Chapter 4 (“Media Risks: Uncertain Journalists”) indicates that social media is the main reason for the prevalence of conspiracy theories and false news (Sharma et al., 2017). More and more people prefer to get news from social media. During the pandemic, people pay close attention to the media for fear of risks. The media will provide deterministic stories to cater to users. Nevertheless, the information may be wrong, resulting in more risk perception and anxiety to the public. Eliminating false news may effectively create a consistent, evidence-based information environment, but regulation may also cause more distrust. To build public trust in the media means that authorities need to communicate with the public openly and transparently, develop communication strategies for different target groups, and guide citizens to think heuristically.

The following three chapters deal with subjective risk perception and mental health. Chapter 5 (“Risk-Perception: Uncertain Interpretations”) explains that although social interaction, culture, government, and globalization impact the risk perception of individuals, their judgment is more based on their subjective explanation. Therefore, health authorities need to publicize the risk of pandemic and guide people to take reasonable ways to deal with it, such as reminding citizens to exercise and actively contacting relatives and friends. Chapter 6 (“Mental Health Risks: Uncertain Minds”) continues explicating the relationship between the pandemic and mental state. Along with the arrival of the pandemic, there are many mental health problems, such as anxiety, depression, and insomnia. The adverse effects of these mental health problems are long-term, making people more vulnerable to infection. Chapter 7 (“Existential Risks: Uncertain Life”) indicates that excessive attention to death and certainty will make people lose the pursuit of other life meanings, thus losing the flexibility of survival. The author proposes that the life of an individual itself is full of uncertainty and threat, and the pursuit of the meaning of life in uncertainty may be the key to rebuild the survival elasticity.

In the last chapter (“The World Resilience Society”), Vos concludes that while the infection rate, mortality, the best coping style, and vaccine effectiveness are uncertain, the only certainty may be uncertainty. To wait for certainty in anxiety or to live in harmony with uncertainty is a question. Uncertainty can bring crisis, but it does not prevent people from living a meaningful and satisfying life in a crisis society (Vos, 2020). Vos summarizes that a world's risk society denies uncertainty or transfers uncertainty to others, while a world's elastic society recognizes uncertainty and regards it as an opportunity.

Throughout the book, Vos analyzes the widespread impact of COVID-19 on both individuals and society and points out that people's misunderstanding of uncertainties leads the vital harm. The author emphasizes that as long as people recognize the uncertainty and establish psychological flexibility, it will be enough to deal with various crises in the future. Entering a flexible world society means treating uncertainty as an opportunity. Unfortunately, some of the views of the author seem to exaggerate the psychological effect. He criticizes mass immunization, active protection, isolation, and other policies during the pandemic but does not give further substantive measures. Nevertheless, while the world is still struggling with COVID-19, some of the ideas put forward in this book are worth deep thinking.

## Author Contributions

JZ and ZL wrote the manuscript, with larger contributions by JZ. Both authors contributed to the article and approved the submitted version.

## Funding

This work was supported by The National Social Science Foundation (20FZXB017) and Fundamental Research Funds for the Provincial Universities of Zhejiang (GK219909299001-204 and GK219909299001-214).

## Conflict of Interest

The authors declare that the research was conducted in the absence of any commercial or financial relationships that could be construed as a potential conflict of interest.

## Publisher's Note

All claims expressed in this article are solely those of the authors and do not necessarily represent those of their affiliated organizations, or those of the publisher, the editors and the reviewers. Any product that may be evaluated in this article, or claim that may be made by its manufacturer, is not guaranteed or endorsed by the publisher.
